# Lateral plate number in low‐plated threespine stickleback: a study of plasticity and heritability

**DOI:** 10.1002/ece3.2020

**Published:** 2016-04-06

**Authors:** Truls H. Hansson, Barbara Fischer, Anna B. Mazzarella, Kjetil L. Voje, Leif Asbjørn Vøllestad

**Affiliations:** ^1^ Department of BiosciencesCentre for Ecological and Evolutionary SynthesisUniversity of OsloPO Box 1066 BlindernN‐0316OsloNorway; ^2^ Department of Theoretical BiologyUniversity of ViennaAlthanstrasse 141090ViennaAustria; ^3^ Konrad Lorenz Institute for Evolution and Cognition ResearchMartinstrasse 12A‐3400KlosterneuburgAustria

**Keywords:** Adaptation, epistasis, evolution, *Gasterosteus aculeatus*, phenotypic plasticity

## Abstract

In the threespine stickleback *Gasterosteus aculeatus* model system, phenotypes are often classified into three morphs according to lateral plate number. Morph identity has been shown to be largely genetically determined, but substantial within‐morph variation in plate number exists. In this study, we test whether plate number has a plastic component in response to salinity in the low‐plated morph using a split‐clutch experiment where families were split in two, one half raised in water at 0 and the other at 30 ppt salt. We find a small salinity‐induced plastic effect on plate number in an unexpected direction, opposite to what we predicted: Fish raised in freshwater on average have slightly more plates than fish raised in saltwater. Our results confirm that heritability of plate number is high. Additionally, we find that variance in plate number at the family level can be predicted from other family level traits, which might indicate that epistatic interactions play a role in creating the observed pattern of lateral plate number variation.

## Introduction

The evolutionary history of the threespine stickleback (*Gasterosteus aculeatus*) is a well‐known example of an adaptive radiation. Saltwater populations of stickleback have independently invaded and adapted to freshwater environments recurrently since the last ice age. One of the most notable phenotypic changes seen in this radiation is the reduction of lateral bony armor plates (Bell and Foster 1994; Lescak et al. 2015). Stickleback are commonly categorized into three plate morph categories: Marine fish are predominantly “fully plated” (>30 plates), fish in brackish water are commonly intermediately or “partially plated”, while freshwater fish usually have <10 plates (“low plated”) (Hagen and Gilbertson 1972; Bell and Foster 1994; Myhre and Klepaker 2009; Song et al. 2010). The *ectodysplasin A* (*EDA*) locus has been identified to determine with about 70% accuracy whether a fish has “full,” “partial,” or “low” armor (Colosimo et al. 2004, 2005; Cresko et al. 2004). It has also been shown that lateral plate number is a heritable trait and that its heritability is high across all three morphs (Hagen and Gilbertson 1973; Hermida et al. 2002; Aguirre et al. 2004; Loehr et al. 2012).

Despite the fact that morph identity is to a large extent genetically determined, there remains substantial within‐morph variation in plate number in these fish; in other words, even for the same *EDA* configuration, large variation in plate number persists (Bell 1984; Colosimo et al. 2004; Nosil and Reimchen 2005). Many different ecological drivers have been suggested to explain this variation (Hagen and Gilbertson 1972; Moodie and Reimchen 1976; Gross 1977; Bell and Foster 1994; Reimchen 2000; Bergstrom 2002; Kitano et al. 2008; Marchinko 2009; Myhre and Klepaker 2009; Spence et al. 2012; Voje et al. 2013), but this body of work does not agree on a single driver, or a combination of drivers, which cause the observed variation in plate number. Some studies even come to opposite conclusions regarding the ecological drivers explaining plate number variation despite using identical populations and investigating the same environmental variables (Spence et al. 2013; MacColl and Aucott 2014). Although there are indications of predation and other drivers having effects on within‐morph plate number, a complete understanding of this variation is clearly still missing (Hagen and Gilbertson 1972; Moodie et al. 1973; Bell and Richkind 1981; Reimchen 1983). This observation led us to ask whether phenotypic plastic‐ity could play a role in plate number variation, thereby contributing to the lack of consensus.

In this experimental study, we aim at contributing to identify proximate reasons for within‐morph lateral plate number variation. To this end, we test whether part of the within‐morph variation is due to phenotypic plasticity. As salinity is an essential environmental factor that varies between environments that are inhabited by the threespine stickleback, we tested for phenotypic plasticity in response to salinity. We designed a split‐clutch experiment in which we reared offspring of low‐plated stickleback in saltwater and freshwater, respectively. We then evaluated the heritability of plate number and assessed patterns of plate number variation within and across families of the low‐plated morph.

## Methods

Threespine stickleback were collected from lake Glitredammen (59.931767°N, 10.498728°E) in June 2013 using minnow traps (Breder 1960). Each gravid female was paired with one mature male, and 16 crosses were made. Each full‐sib family was split into two after hatching. One half was reared in a separate tank in saltwater (25–30 ppt) and the other half in a separate freshwater tank (0 ppt) (one half of the full‐sib clutch is hereafter referred to as a half‐clutch). A total of 917 fish were allocated to the treatments.

The experiment was terminated after 5 months, when all fish had grown to at least 30 mm in body length, as this is when lateral plates are assumed to be fully developed (Bell 1981; Banbura 1987). Fish were euthanized using benzocaine, and body length was measured. See Mazzarella et al. (2015) for a full description of experimental design and protocols.

Fish were stained in Alizarin Red, and lateral plates were counted on both sides for each offspring and parent. Lateral plates were recounted for 96 fish to estimate counting error. Plate counts were highly repeatable (*r *=* *0.97 for right side and *r *=* *0.98 for left side).

All parents were confirmed as homozygotes for the low‐plated *EDA*‐allele (low‐plated morph) by amplification and gel electrophoresis of the microsatellite Stn 382 (Colosimo et al. 2005).

Heritability was estimated by parent–offspring regression. The mean of the total plate number (sum of plates on both sides of the fish) for each clutch was regressed on the mid‐parental total plate number (mean of the parents) and narrow‐sense heritability *h* ^2^ was estimated as the slope of the regression.

We tested for salinity‐induced plasticity of lateral plate number using general linear mixed models using a combination of treatment, density, and fish size as explanatory variables. Family was included as a random effect to account for nested family effects. Fish size was included to assess whether plate number was associated with overall size. We also included density of fish in individual tanks as body size is expected to correlate with density as a consequence of density‐dependent growth. The different models were compared using the Akaike information criterion (AIC) (Akaike 1974).

Next, we analyzed plate number variance at the family level. First, to determine which fraction of the total variance in plate number was within‐family versus between‐family, we conducted an ANOVA. Second, we tested whether plate number variance of each half‐clutch was explained by other family‐level traits using a general linear model. For this purpose, the variance in plate number for each half‐clutch was modeled as a function of the mid‐parent plate number, plate number mean of each half‐clutch, and the interaction of these two variables. Treatment was included as a random effect.

All statistical analyses were conducted in the statistical environment R version 3.0.1 (R Core Team 2013, Vienna, Austria, http://www.R-project.org/) using standard linear models and the packages lme4 and nlme (Pinheiro et al. 2007; Douglas et al. 2015).

## Results

At the end of the experiment, lateral plate number was counted for 738 offspring fish from the 16 families. In addition to 32 parents, 357 offspring fish were counted from the freshwater treatment group and 381 fish from the saltwater treatment group. An average of 22.3 [8.3] (mean [standard deviation, SD]) fish were counted from each freshwater tank (half‐clutch) and 23.8 [8.0] fish from each saltwater tank. Survival was high in both treatments across all families (mean [SD]: freshwater: 0.8 [0.1], saltwater: 0.8 [0.1]).

**Figure 1:**
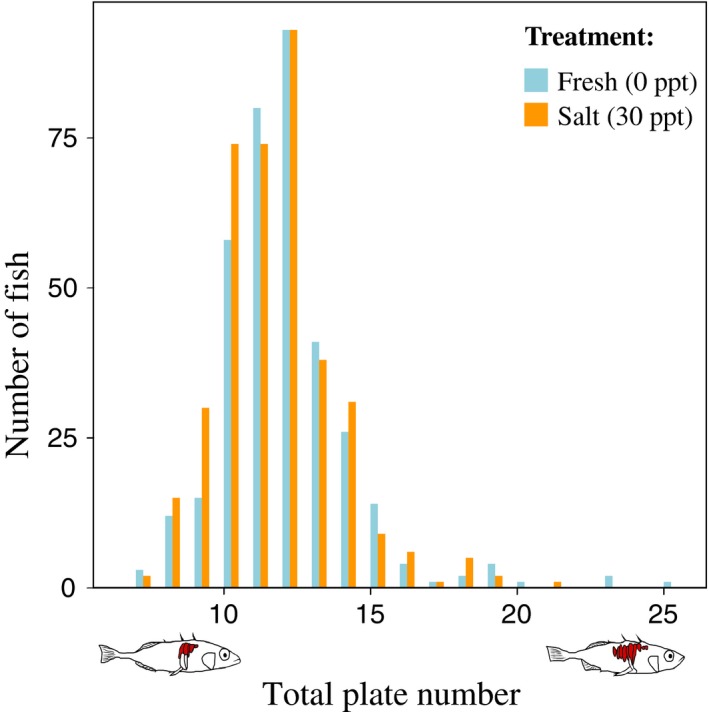
Plate number distributions for the two treatments for all offspring from all families.

**Figure 2:**
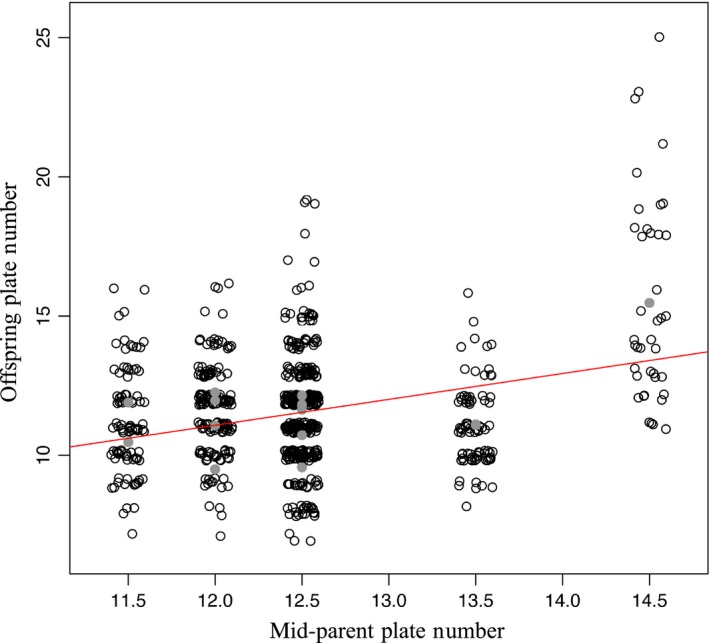
Parent–offspring regression. Shown is the linear regression (red line) of mean offspring plate number (filled gray circles) on mid‐parent total plate number, calculated as mean of the parents, for the 16 families. Mean offspring plate number is the mean of the total plate number (both sides) of all offspring in a family. Individual offspring plate numbers (open black circles) are shown together with the family means. Noise was added to the individual plate number counts and to the corresponding mid‐parent plate numbers for better visualization, to avoid overlap. Heritability *h* ^2^ was estimated as the slope of the regression line, which was 0.93.

Plate number variation was larger among the offspring fish than among the parents (mean [SD]: freshwater: 11.8 [2.3], saltwater: 11.5 [2.0], see Fig. 1; parents: 12.5 [1.2], see also Fig. 2).

Narrow‐sense heritability of total plate number was estimated to be large and significantly greater than zero (*h* ^*2*^ **=* *0.93, SE* *=* *0.40, *P = *0.035, df =* *14, Fig. 2). Note, however, that the standard error (SE) of this estimate is large.

Average size did not differ between treatments (mean [SD]: freshwater: 35.5 [2.9] mm, saltwater: 35.8 [2.9] mm), but average size decreased with increasing density in both freshwater (correlation *r *=* *−0.16, *P *=* *0.003) and saltwater (*r *=* *−0.33, *P *<* *0.0001) treatments.

The model with salinity as the only explanatory variable and family as random variable explained variation among individuals in total lateral plate number best according to AIC. Based on this model, fish raised in 25–30 ppt saltwater are expected to have on average 0.31 (SE* *=* *0.13) fewer plates than those raised in 0 ppt freshwater. The second best model differed by 2.06 AIC units and included an additive effect of density, but the effect of salinity was still significant and similar to that for the best‐fitting model. Fish size, independent of density, had no effect on plate number.

Next, we assessed variation in total lateral plate number within and across families. Family identity explained 29% of the total variance in plate number while 71% was within‐family variance (one‐way ANOVA).

**Figure 3:**
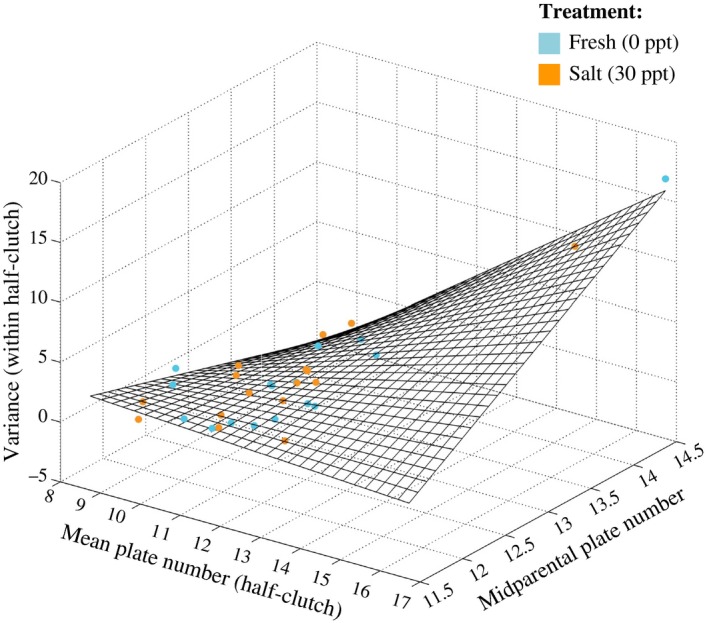
Statistical model fit for within‐family variation in plate number, shown on the vertical axis, modeled as a linear function of mid‐parental plate number, mean within‐half‐clutch plate number, and their interaction. The model surface (black mesh) is shown together with the data. The slope and interaction coefficients of the fitted model were −9.85 (SE **=* *1.98) for mid‐parental plate number, −10.26 (SE **=* *2.09) for half‐clutch mean plate number, and 0.88 (SE **=* *0.16) for their interaction.

As expected, variance in plate number in a clutch increased with both increasing mean half‐clutch plate number (*r *=* *0.78) and with increasing mid‐parental plate number (*r *=* *0.58). The linear model including mid‐parent plate number, mean half‐clutch plate number, and their interaction explained within‐clutch variation in plate number very well (*r* ^2^ **=* *0.85) (Fig. 3). Both explanatory variables and their interaction were statistically significant (*P *<* *0.001 for all three coefficients). This result was robust with respect to pooling the two treatment groups in each full‐sib family (*r* ^2^ **=* *0.88) and for including a random factor for treatment (estimated random intercepts did not differ between treatments, compare Fig. 3).

## Discussion

We tested in a split‐clutch experiment whether lateral plate number of the low‐plated morph of the threespine stickleback is plastic in response to salinity. We found a small effect of salinity on average plate number, in the unexpected direction that fish raised in saltwater had on average 0.31 fewer plates than fish raised in freshwater. To our knowledge, this is the first empirical study testing for plasticity in plate number in the threespine stickleback. In agreement with earlier studies, we found substantial heritability of lateral plate number, *h* ^*2*^ **=* *0.93 (previous estimates of *h* ^*2*^: 0.90 (Aguirre et al. 2004), 0.84 (Hagen and Gilbertson 1973), 0.46 (Loehr et al. 2012), and 0.37 (Hermida et al. 2002)). Furthermore, our assessment of variation in plate number at the family level indicates that nonadditive genetic effects might play a role in the determination of lateral plate number.

Despite the fact that we identified a plastic effect of salinity on plate number, we want to caution against an over‐interpretation of this finding. The effect that we identified is small and was probably only recognized because our sample size was so large (*n* = 738). However, it is interesting that M. Wund and colleagues at the Biology Dept. of the College of New Jersey have found salinity‐induced plasticity in plate height in the same direction: An increase in salinity causes a plastic decrease in plate height in fish of freshwater origins (pers. comm.). That the direction of change in our experiment is in the opposite direction as compared to the adaptive pattern found in nature is puzzling. What we found could be a nonadaptive reaction to the stress of living in a non‐native salinity. There is also the possibility that an even larger effect was masked by maternal effects, as the female parents were all collected and kept in freshwater until the crosses were made, and as such the egg cytoplasm salt content was likely affected by this. Perhaps allowing the parental females to acclimate to freshwater and develop their clutches in the experimental salinity would show us an increased or otherwise different effect.

We also tested whether variance in plate number at the family level was associated with other family‐level traits and found that a general linear model with mid‐parental plate number, mean plate number, and their interaction as explanatory variables explained 85% of the variation in lateral plate number variance within‐half‐clutches. This result is in line with Colosimo et al. (2005), who found multiple loci that influence within‐morph plate number distribution. The significant interaction effect is surprising; however, this suggests that the loci responsible for plate number determination in these fish may interact in nonadditive ways, that is, that there are across‐locus interactions (epistasis). This interpretation is based on the assumption that our data represent the true lateral plate number distribution in the population.

Both the results that heritability of plate number is high and the relevant interaction effect in explaining plate number variance, which hints at epistasis, depend on inclusion of family 15 in the sample. This family had a mid‐parental plate number of 14.5, which was higher than for the other families (11.5–13.5) (see Fig. 2). Family 15 was, like all other families, identified as homozygous for the low‐plated EDA‐allele and it consisted of 40 offspring individuals, which is a substantial family size. We therefore found no reason for excluding this family from the analysis. However, for completeness, we repeated our analysis without family 15. Exclusion of family 15 makes the heritability estimate for plate number drop to zero, which is inconsistent with previously reported estimates (Hagen and Gilbertson 1973; Hermida et al. 2002; Aguirre et al. 2004; Loehr et al. 2012). The interaction effect in the model for family‐level variance in plate number disappears when family 15 is excluded.

The substantial lateral plate variation we observe in our study population could indicate that selection has not been sufficiently strong or effective to fix alleles in non‐*EDA* loci that control the number of plates in this population. An alternative and nonmutually exclusive interpretation is that differences in fitness might be minimal for a broad range of plate numbers within the low‐plated morph in this population. This is a possible scenario if the number of lateral plates is not the primary reason for a fitness advantage of the low‐plated *EDA* genotype in freshwater. The rapid loss of lateral plates in stickleback might then instead be caused by indirect selection through pleiotropic effects of the *EDA* gene on one or more other traits that are under directional selection (Barrett et al. 2009). Results in line with this hypothesis were presented in a study by Le Rouzic et al. (2011), who found a much larger fitness advantage of the low‐plated *EDA* genotype that often (but not always) produces low‐plated stickleback compared to individuals with an actual low‐plated phenotype in a freshwater pond. If the high variation in plate number within the low‐plated morph is neutral, this might explain as to why there is so little evidence for specific ecological variables driving lateral plate evolution. Although predation seems to play a role as an agent of selection of lateral plates (e.g., Reimchen 2000; Bergstrom 2002; Marchinko 2009), the detailed nature of the selective forces and the causes of plate reduction in stickleback in freshwater systems remain to be fully explained.

## Data Accessibility

Comma‐separated values file with fish ID, family, treatment, and plate count is available on Dryad (doi:10.5061/dryad.jm2m1).

## Conflict of Interest

None declared.
